# Vulvar Paget disease secondary to high-grade urothelial carcinoma with underlying massive vascular embolization and cervical involvement: case report of unusual presentation

**DOI:** 10.1186/s13000-019-0895-0

**Published:** 2019-11-07

**Authors:** Walquiria Quida Salles Pereira Primo, Guttenberg Rodrigues Pereira Primo, Dunya Bachour Basilio, Karime Kalil Machado, Jesus Paula Carvalho, Filomena M. Carvalho

**Affiliations:** 1 Department of Gynecology, Faculdade de Medicina, Universidade de Brasília, Brasília, DF Brazil; 2 Gynecology Service, Hospital Regional da Asa Norte (HRAN), Secretaria de Estado da Saude, Brasilia, DF Brazil; 3 Department of Pathology, Instituto Hospital de Base do Distrito Federal IHBDF, Secretaria de Estado da Saude, Brasilia, DF Brazil; 4 Medical Oncology Service, Hospital Sírio Libanês, Brasília, DF Brazil; 5 Instituto do Cancer do Estado de São Paulo ICESP, Faculdade de Medicina, Universidade de São Paulo, Sao Paulo, SP Brazil; 6 Department of Pathology, Faculdade de Medicina FMUSP, Universidade de São Paulo, Av. Dr. Arnaldo, 455 – room 1149, Sao Paulo, SP 01246-903 Brazil

**Keywords:** Extramammary Paget disease, Urothelial carcinoma, Vulva, Lymphatic embolization

## Abstract

**Background:**

Vulvar extramammary Paget disease is a rare chronic condition, that presents with non-specific symptoms such as pruritus and eczematous lesions. Because most of these lesions are noninvasive, the distinction between primary and secondary Paget disease is crucial to management.

**Case presentation:**

We report an unusual case of vulvar Paget disease associated with massive dermal vascular embolization, cervicovaginal involvement and metastasis to inguinal and retroperitoneal lymph nodes. The intraepithelial vulvar lesion had a classical appearance and was accompanied by extensive component of dermal lymphovascular tumor emboli, similar to those observed in inflammatory breast carcinoma. Immunohistochemical analysis revealed that the lesion was secondary to high-grade urothelial cell carcinoma. The patient had a history of superficial low-grade papillary urothelial carcinoma of the bladder, which had appeared 2 years before the onset of vulvar symptoms.

**Conclusions:**

Eczematoid vulvar lesions merit careful clinical examination and biopsy, including vulva mapping and immunohistochemistry. The information obtained may help to define and classify a particular presentation of Paget disease. Noninvasive primary lesions do not require the same aggressive approaches required for the treatment of invasive and secondary disease.

## Background

Extramammary Paget disease (EMPD) is a rare condition that manifest as cutaneous neoplasms, usually found in areas with high-density apocrine glands, such as the female and male genital areas [1]. In women, the most frequent localization of the EMPD in women is the vulva (65% of the cases), accounting for only 1 to 2% of the neoplasms arising in the anogenital area [2]. The condition is more common in postmenopausal Caucasian women. Mean age at the time of diagnosis is 65 yr [2].

Vulvar EMPD can be subdivided as primary (cutaneous) and secondary (non-cutaneous) types [1]. Primary EMPD originates from intraepithelial cells of the epidermis or skin appendages and can rarely present focal invasion through the basal membrane or association with a vulvar adenocarcinoma. About 20% of cases of primary EMPD arise from the skin of the perianal region [3]. Secondary EMPD is a neoplastic involvement of the vulvar skin by a non-cutaneous neoplasm, through by epidermotropic metastasis or direct extension.

Here, we present an unusual case of EMPD of the vulva, secondary to high-grade urothelial carcinoma, that presented 2 yr after the patient was diagnosed with low-grade bladder urothelial carcinoma, associated with massive dermal lymphatic carcinomatosis, cervical involvement, and lymph node metastasis.

## Case representation

A Caucasian woman, 59 yr of age, was referred to our institution in September, 2017 with a chief complaint of severe vulvar pruritus that began 6 yr earlier, with a drastic deterioration in the patient’s condition over the past 2 years. The patient had made use of several topical medications, without result. No complaints of vaginal discharge or urinary symptoms were noted.

The patient’s medical history revealed that she had been a smoker from the age of 15 to 51 yr, consuming one pack of cigarettes per day. In 2009, the patient developed a superficial non-muscle invasive low-grade papillary urothelial carcinoma of the bladder. She was treated with transurethral resection followed by intravesical mitomycin. In 2010, the patient presented with local recurrence. The lesion was endoscopically resected. The patient received 1 yr of treatment with full-dose bacillus Calmette-Guérin (BCG) intravesical immunotherapy (induction plus once weekly instillations every 3 weeks at 3, 6, and 12 mo). The patient was followed up by a urologist, and her recovery was uneventful.

**Fig. 1:**
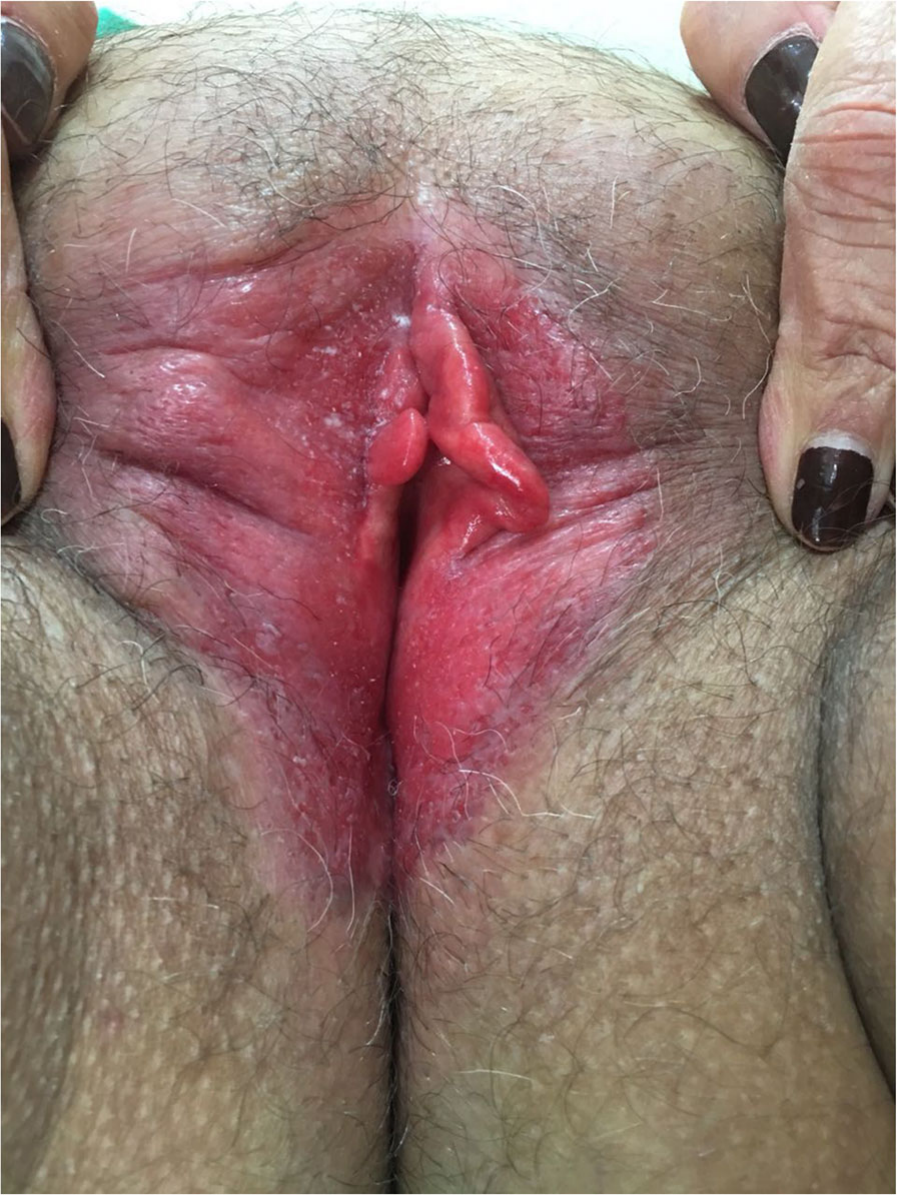
Extensive erythematous desquamative lesion involving major and minor labia bilaterally and perineal and perianal regions

A physical examination was performed when the patient arrived at our institution in 2017. The examination revealed an erythematous, flat, scaly lesion, with poorly defined borders, located bilaterally in labia majora and minora. The lesion extended to the vestibular, perineal, and perianal regions (Fig. 1). The patient’s lymph nodes were not palpable.

The results of punch biopsy revealed the presence of non-invasive vulvar EMPD, as confirmed by immunohistochemistry (positive for cytokeratin 7 (CK7), negative for S-100 protein). Cystoscopy, colonoscopy, bilateral mammography, and chest tomography showed no evidence of any suspicious lesion.

The patient received imiquimod 5% immunotherapy for 6 mo. This treatment failed to improve the patient’s symptoms and vulvar pain. On July 21, 2018, a simple vulvectomy was performed to excise all visible lesions. A cervicovaginal smear was collected for cytological examination and human papillomavirus (HPV) DNA study by real-time polymerase chain reaction (PCR) assay.

**Fig. 2:**
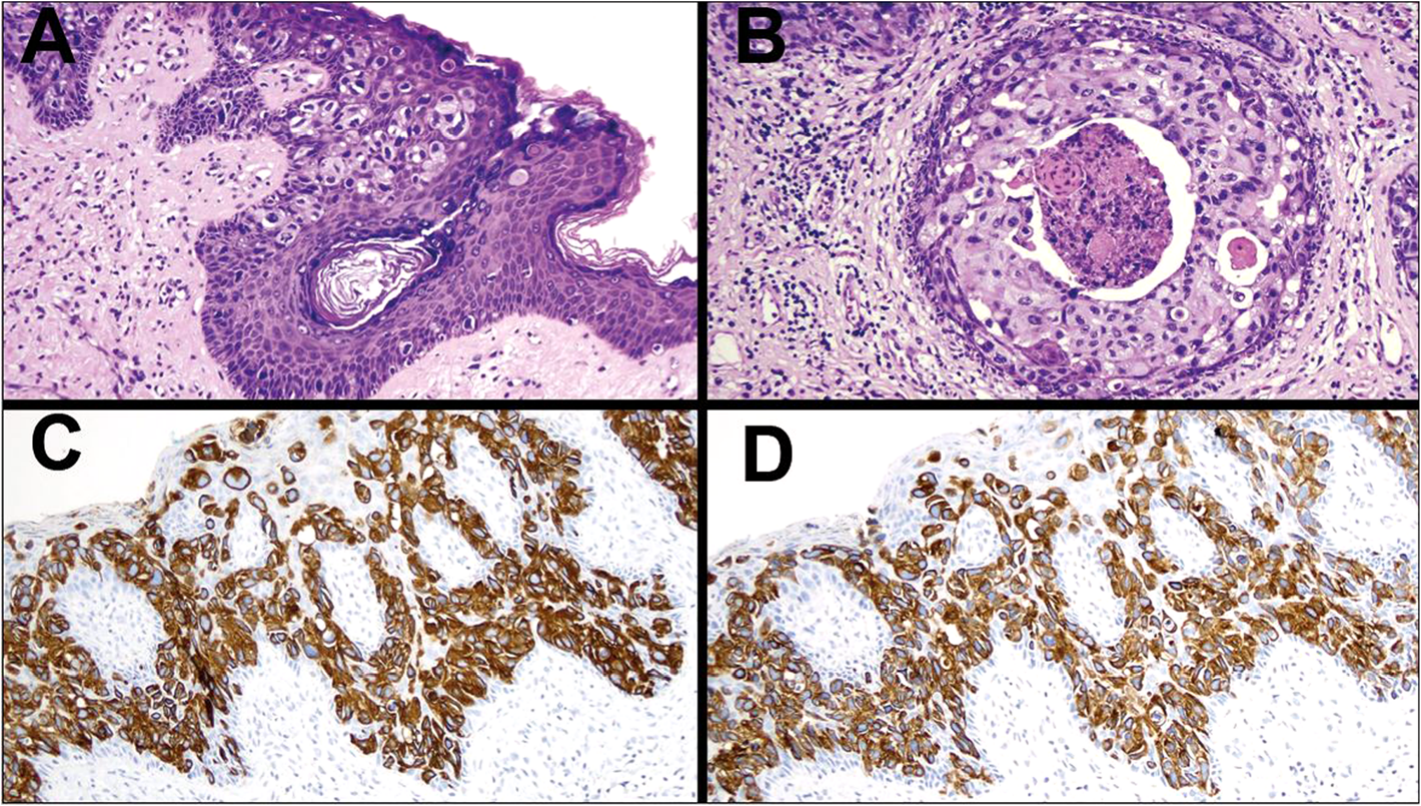
Extramammary Paget disease characterized by large pleomorphic cells in epidermis (**a**) and duct of cutaneous appendage showing comedonecrosis (**b**), positive to cytokeratin 7 (**c**) and cytokeratin 20 (**d**)

**Fig. 3:**
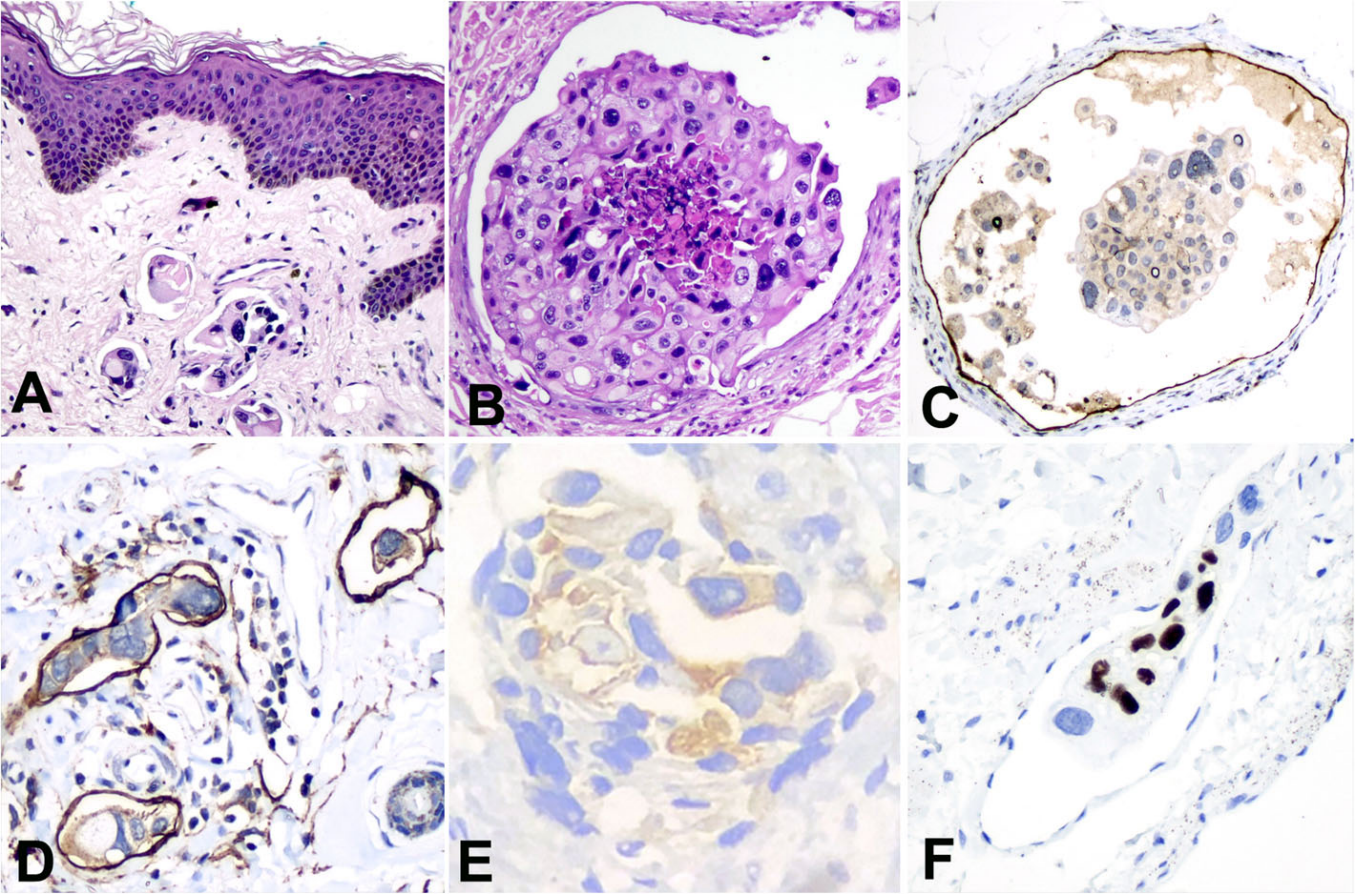
Dermal embolization in extramammary Paget disease. **a** Emboli with neoplastic cells in hematoxylin-eosin histological section. **b** Distended dermal vessel with neoplastic cells within. **c** Same vessel of B with endothelial cells stained by D2–40. **d** Dermal vessels stained by WT-1 showing negative neoplastic cells within. **e** Neoplastic cells stained by uroplakin III in a vessel. **f** Neoplastic cells in dermal vessel positive to p63

The entire vulvar skin was submitted for histological examination. Pathological analysis revealed a diffuse intraepidermal proliferation of large cells, with pleomorphic nuclei, distributed throughout the entire thickness of epidermis and extending to the skin appendages (Fig. 2a). The neoplastic cells were numerous, in large groups, distending the appendage ducts, where they were associated with foci of comedonecrosis (Fig. 2b). Intense lymphocytic infiltration could be seen under the epidermis. The dermis and hypodermis showed diffuse neoplastic vascular embolization (Fig. 3a and b). Immunohistochemical study revealed tumor cells positive for cytokeratin 7 (CK7) (Fig. 2c), cytokeratin 20 (CK20) (Fig. 2d), p63 (Fig. 3f), epithelial membrane antigen (EMA), uroplakin III (Fig. 3e), and GATA-3. Tumor cells were negative for gross cystic disease fluid protein (GCDFP-15), estrogen receptor, progesterone receptor, HER2, CDX-2, carcinoembryonic antigen (CEA), and Wilms Tumor 1 (WT1) (Fig. 3d). Staining for p16 was heterogenous for p16. Endothelial cells stained positively for D2–40, highlighting the vessels with negative neoplastic cells within (Fig. 3c).

**Fig. 4:**
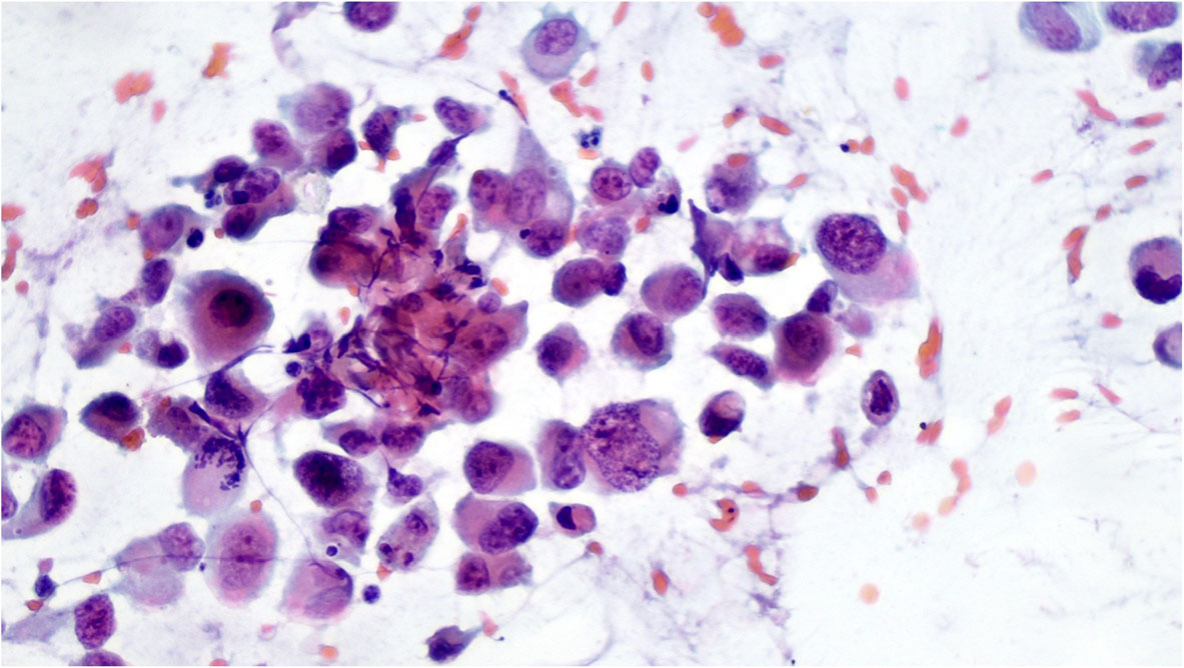
Cervical smear showing large pleomorphic cells, similar to Paget cells (Papanicolaou stain)

This immunohistochemical profile indicated that the Paget cells were of high-grade urothelial carcinoma type. Analysis of the cervicovaginal smear revealed numerous large and atypical cells, similar to those observed in vulvar neoplasia (Fig. 4). The HPV/DNA test was negative.

At this time, the patient had persistent dysuria, lower abdominal pain, and urinary frequency. She was re-evaluated for urological and systemic disease. Cystoscopy demonstrated no macroscopic abnormalities, and the biopsy failed to provide evidence of carcinoma. Pelvic magnetic resonance imaging (MRI) revealed diffuse thickening of the cervical walls with low-signal intensity on T2-weighted sequences, heterogeneous enhancement, and restricted diffusion. This thickening extended to the lateral uterine walls, the upper third of the vagina, and the vesicouterine pouch. Positron emission tomography (PET) with fluoro-2-deoxyglucose (FDG) and computed tomography (CT) revealed intense metabolic activity, suggestive of neoplastic involvement, in this lesion and in inguinal, pelvic, and para-aortic lymph nodes (measuring up to 11.0 mm in length). The final diagnosis was of metastatic occult high-grade urothelial carcinoma associated with secondary vulvar EMPD. The patient was submitted to standard first-line palliative chemotherapy for urothelial cancer with gemcitabine and cisplatin. She completed the sixth cycle with a partial response according to the RECIST criteria.

## Discussion

EMPD is a very heterogeneous entity regarding etiology, although the clinical and pathological findings of the non-invasive lesion are similar. Management requires careful clinical evaluation and a precise histopathological diagnosis based on the results of immunohistochemistry. The first challenge is to differentiate EMPD from mimics, such as Bowen disease and melanoma, both of which appear as large cells between squamous epidermal cells on routine hematoxylin-eosin staining. On immunohistochemistry, Paget cells express low molecular weight cytokeratins, while squamous cells express high molecular weight cytokeratins. Melanoma cells stain for melanocytic immunohistochemical markers such as HMB45, Melan-A, and S100 protein. Vulva mapping with multiple biopsies is indicated to rule out invasion.

The distinction between primary and secondary EMPD is crucial to management. Primary EMPD without invasion should be treated more conservatively, with less morbidity, with topic imiquimod 5% cream immunotherapy [4, 5]. In primary EMPD, Paget cells are positive to CK7, GCDFP-15, and GATA-3. This profile of immunohistochemical expression is similar to that of breast tissue, a testimony of the frequent origin in anogenital mammary-like glands. At least some of primary EMPD may originate from Toker cells, a population of intra-epidermal CK7-positive glandular cells, corresponding a probable component of excretory ducts of anogenital mammary-like glands [6]. Similar to mammary Paget disease, most EMPD are positive for HER2, indicating the possibility that anti-HER2 therapy may be effective for the treatment of invasive and/or metastatic EMPD [7].

Although the rate of local recurrence is high in all patients with EMPD (approximately 40%), the risk of other malignancies is limited to patients with invasive disease (8% of cases) and patients with secondary EMPD [8–10]. Most cases of EMPD are primary and non-invasive. In a cohort study conducted by van der Linden et al. that included 199 patients, 76.9% of EMPD cases were primary noninvasive [9]. Immunohistochemical analysis may aid in identifying secondary EMPD. Primary lesions are CK7+/CK20-; lesions with intestinal phenotype are CK7−/CK20+/CDX2+; lesions with urothelial phenotype are CK7+/CK20+/uroplakin+ [8]. EMPD of urothelial origin is the rarest type of secondary EMPD, and urothelial neoplasia can appear before or up to 13 yr after the onset of vulvar symptoms [11–13]. Identification of urothelial phenotype is fundamental to screening and following the patient to monitor for concurrent or future malignancy [11]. Padhy et al. presented a case of secondary Paget disease of urothelial origin without previous or concurrent urinary tract malignancy. Urothelial origin was determined based on the immunohistochemical profile, with expression of GATA3, uroplakin III, and p63, similar to our case and to other authors [11, 14]. In our case, the previous urothelial carcinoma was of low grade and had been under control for years. The possibility of recurrence with a higher grade phenotype should not be ignored, nor should the possibility that the current vulvar lesion is secondary to another occult or even future urothelial neoplasia.

An interesting feature of the case reported here was the cervical involvement apparent on cervicovaginal cytology. Koyanagi et al. presented a case of high grade urothelial carcinoma with pagetoid spread to the vagina, without accompanying Paget disease presentation [14]. The authors discuss the differences between pagetoid urothelial carcinoma, pagetoid colorectal carcinoma and extramammary Paget disease, highlighting the importance of cervical screening cytology.

In addition to the urothelial phenotype, the case presented here had massive underlying vascular involvement, similar to that observed in patients with inflammatory breast carcinoma. Murata et al. described 6 cases of EMPD, one of them vulvar, with lymphatic infiltration of the skin and a distinct erythema of the inguinal and genital regions (area covered by underpants) [15]. All patients with the “underpants-pattern erythema” died of the disease, with mean survival of 13 mo. Although our case does not fit the criteria of the “underpants-pattern erythema” as described by Murata et al., it presented the same extent of vascular involvement, confirming that this finding configures important sign of advanced and aggressive disease. More recently, Maeda et al. described an autopsy case series that included 7 cases of advanced EMPD, all of which primary from genital area and with the so-called underpants erythema [16]. All the cases presented multiple lymph node metastases, as well as liver and lung metastases associated with multiple extensive lymphovascular spaces that were plugged with Paget cells. Because of the predominance of vascular involvement as opposed to nodule formation, CT images were not helpful in determine the extent to which the disease has spread [16]. The authors highlight the importance of vascular involvement in the progression of disease. Future in vitro studies should investigate a possible role of vascular mimicry in this type of presentation, which, as we observed in our case, bears some resemblance to inflammatory breast carcinoma.

## Conclusion

Careful clinical and pathological examination, including immunohistochemistry, is mandatory for defining and classifying Paget disease. More aggressive approaches must be directed to invasive, secondary, and the rare manifestations of EMPD that are accompanied by vascular embolization.

## Data Availability

Yes

## Abbreviations

CK: Cytokeratin
EMPD: Extramammary Paget disease
FDG-PET/CT: [18F]-fluorodeoxyglucose (FDG) positron emission tomography-computed tomography (PET/CT)
GCDFP-15: Gross cystic disease fluid protein 15
MRI: Magnetic Resonance Image
RECIST: Response Evaluation Criteria in Solid Tumours
